# Recognition and binding of mismatch repair proteins at an oncogenic hot spot

**DOI:** 10.1186/1471-2199-6-6

**Published:** 2005-03-14

**Authors:** Michael Edelbrock, Huiling He, Allen Schroering, Martha Fernstrom, Sangeetha Bathala, Kandace J Williams

**Affiliations:** 1 Department of Biochemistry & Cancer Biology, Medical College of Ohio, Toledo OH, USA; 2 Division of Human Cancer Genetics, The Ohio State University, Columbus OH, USA; 3 Department of Cell Biology and Neuroscience, University of South Alabama, Mobile AL, USA

## Abstract

**Background:**

The current investigation was undertaken to determine key steps differentiating G:T and G:A repair at the H-*ras *oncogenic hot spot within the nuclear environment because of the large difference in repair efficiency of these two mismatches.

**Results:**

Electrophoretic mobility shift (gel shift) experiments demonstrate that DNA containing mismatched bases are recognized and bound equally efficiently by hMutSα in both MMR proficient and MMR deficient (*hMLH1-/-*) nuclear extracts. Competition experiments demonstrate that while hMutSα predictably binds the G:T mismatch to a much greater extent than G:A, hMutSα demonstrates a surprisingly equal *ratio *of competitive inhibition for both G:T and G:A mismatch binding reactions at the H-*ras *hot spot of mutation. Further, mismatch repair assays reveal almost 2-fold higher efficiency of *overall *G:A repair (5'-nick directed correct MMR to G:C and incorrect repair to T:A), as compared to G:T overall repair. Conversely, *correct *MMR of G:T → G:C is significantly higher (96%) than that of G:A → G:C (60%).

**Conclusion:**

Combined, these results suggest that initiation of *correct *MMR requires the contribution of two separate steps; initial recognition by hMutSα followed by subsequent binding. The 'avidity' of the binding step determines the extent of MMR pathway activation, or the activation of a different cellular pathway. Thus, initial recognition by hMutSα in combination with subsequent decreased binding to the G:A mismatch (as compared to G:T) may contribute to the observed increased frequency of incorrect repair of G:A, resulting in the predominant G**G**C → G**T**C (Gly → Val) *ras-*activating mutation found in a high percentage of human tumors.

## Background

Several different DNA repair systems have evolved within all living cells to correct mispaired or damaged nucleotide residues generated either by endogenous events or by exposure to exogenous mutagenic agents [1,2]. The frequency of mutational events varies widely within the genome, and specific sites harboring increased frequency of mutation are now defined as 'hot spots' of mutation. The human *ras *protooncogene family contains three such hot spots – codons 12, 13, and 61. Factors contributing to these and other hot spots of mutation are still largely unknown, despite much investigation, but now appear to have several different contributions, such as type of DNA damage, genomic location, surrounding sequence, cell cycle position, efficiency of the optimal DNA repair pathway, and involvement of alternate repair and other cellular pathways.

DNA mismatch repair (MMR) is a repair system that corrects mispaired nucleotides and insertion/deletion loops (IDLs), resulting from replication, recombination, or repair errors. Consequences of defects in this DNA repair pathway are evidenced by microsatellite instability (MSI), elevated mutation frequency throughout the genome, enhanced recombination events, as well as tolerance to cytotoxic effects of alkylating agents as evidenced by decreased apoptosis. Deficient MMR is, in turn, associated with hereditary nonpolyposis colorectal cancer (HNPCC), as well as other types of sporadic tumors in humans and animal models [3-6]. Although the contribution of MMR to highly mutable genomic sites other than microsatellite sequences is largely unknown, several specific genetic mutations involved in neoplastic progression, including *ras-*activating mutations, have been reported as frequent occurrences in HNPCC and other MSI tumors [7,8].

DNA MMR is conserved amongst highly divergent species, reflecting the essential role of this DNA repair process [9,10]. The MMR system in eukaryotes is more complex and has different mismatch-specific repair efficiencies than that of *E. coli *[11-13]. Several human homologs of bacterial MutS and MutL proteins have now been identified [14-19]. The primary human homologs for MutS that play instrumental roles in MMR include hMSH2, hMSH6, and hMSH3 [20,21]. The hMutSα heterodimer (hMSH2 and hMSH6) has been demonstrated to recognize and bind DNA mispairs and short IDLs [14,21-24]. The hMutSβ heterodimer (hMSH2 and hMSH3) preferentially recognizes and binds IDLs of up to 12 nucleotides. Human cells lacking hMSH2 protein expression also lack its cognate partners, hMSH3 and hMSH6 (due to decreased stability). This lack is associated with defective MMR, microsatellite instability, and is associated with a high percentage of HNPCC [25]. MutL homologs that are most relevant to human MMR are hMLH1 and hPMS2, which form the hMutLα heterodimer [9,26]. Similar to the observed instability of individual MutS homologs, lack of hMLH1 protein expression results in the lack of hPMS2 protein, which in turn results in microsatellite instability, defective MMR, and is also associated with a high percentage of HNPCC [9,10,15,25,27-32]. The MutLα heterodimer is thought to act as a molecular matchmaker between the MutSα-DNA complex and downstream enzyme activities responsible for subsequent identification, excision, and replacement of the incorrect base [9,19]. Biochemical interactions and genetic studies have further implicated proliferating cell nuclear antigen (PCNA), exonuclease I (EXO1), replication protein A (RPA), replication factor C (RFC), and DNA polymerase δ as active participants in the MMR pathway [19,27,29,30,33-35]. Recently, the differential requirement of specific proteins associated with MMR have been identified for both 3'-nick directed and 5'-nick directed MMR by the use of an *in vitro *model [35,36].

We have previously demonstrated that the efficiency of correct mismatch repair within the cell can differ significantly, depending on exact type of mismatch, site-specific location, phase of cell cycle, or cell type [23,24,37,38]. Within this report, we have focused on a more precise understanding of the differences between MMR protein interactions with a G:A (least repaired) or G:T (best repaired) mismatch located at H-*ras *codon 12 to better understand molecular events leading to activating mutations at this site. Our current results indicate that initial recognition and subsequent binding for repair signaling by hMutSα may be two separably measurable steps in the MMR pathway that can significantly affect downstream cellular events.

## Results

### Specificity of hMutSα protein binding to DNA mismatches at H-ras codon 12

**Figure 1:**
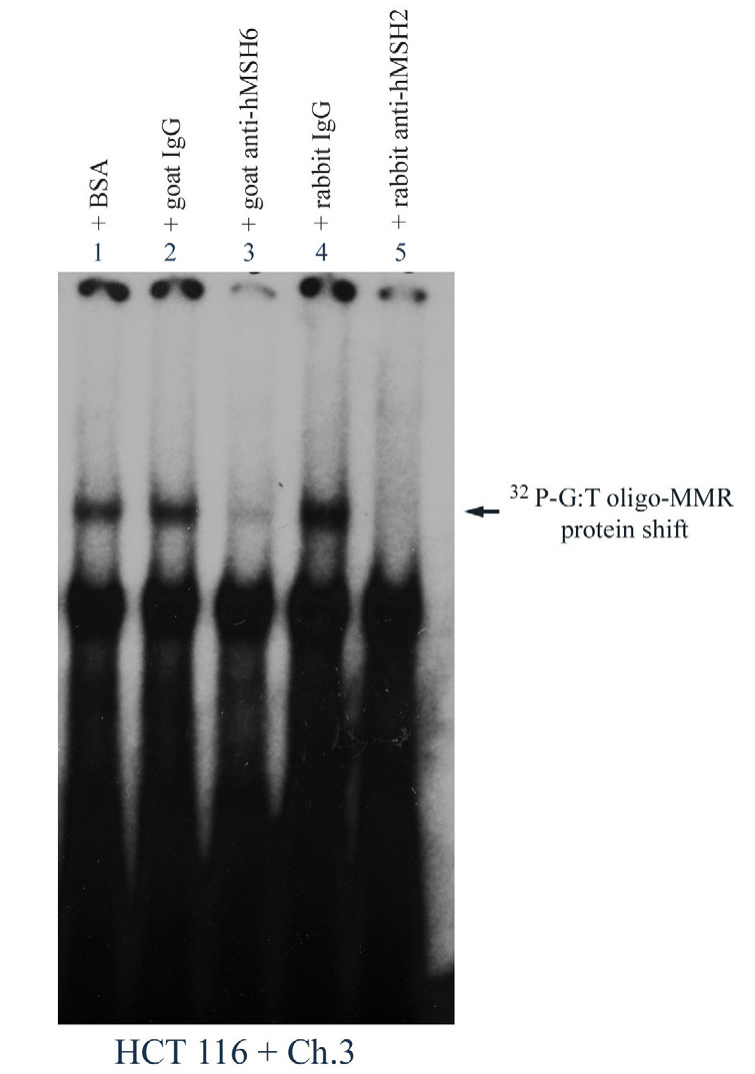
*Specific interaction of hMSH6 and hMSH2 with site-specific mismatched DNA at H-ras codon 12*. Protein-DNA binding reactions and gel shifts were performed using nuclear extracts from HCT 116 + Ch. 3 and equal cpm of [^32^P]-G:T-oligo (69-mer) in the presence of 100X molar excess of cold (unlabeled) homoduplex (G:C). BSA, goat-, or rabbit-nonspecific IgG (lanes 1, 2, 4 respectively), goat anti-hMSH6 (lane 3), and rabbit anti-hMSH2 (lane 5) were also included in the binding reactions as indicated. The lower gel shift band in each lane is due to biotin end-labeling of the probe.

To determine that the binding complex recognizing mismatches at codon 12 of H-*ras *in HCT116 + Ch.3 nuclear extract is composed of hMutsα, DNA binding reactions were conducted using [^32^P]-G:T oligos incubated with nuclear extracts from these MMR proficient cells. In confirmation of hMutSα binding to a G:T mismatch at this oncogenic site, the hMutSα :DNA complex was efficiently interrupted by goat anti-hMSH6 and with rabbit anti-hMSH2 (Figure 1, lanes 3 and 5), but not by BSA, nonspecific goat IgG, or nonspecific rabbit IgG (lanes 1, 2, and 4, respectively). As further evidence of specific hMutsα:DNA mismatch binding, addition of 1.5 mM ATP completely disrupted the gel shifted band (results not shown) [24,30]. Thus, MMR protein binding to a G:T mismatch located at H-*ras *codon 12 within the nuclear environment appears to be primarily, if not exclusively, hMutSα.

### Comparison of hMutSα binding 'avidity' to a G:T mismatch located at H-ras codon 12 within MMR competent and deficient nuclear extracts

**Figure 2:**
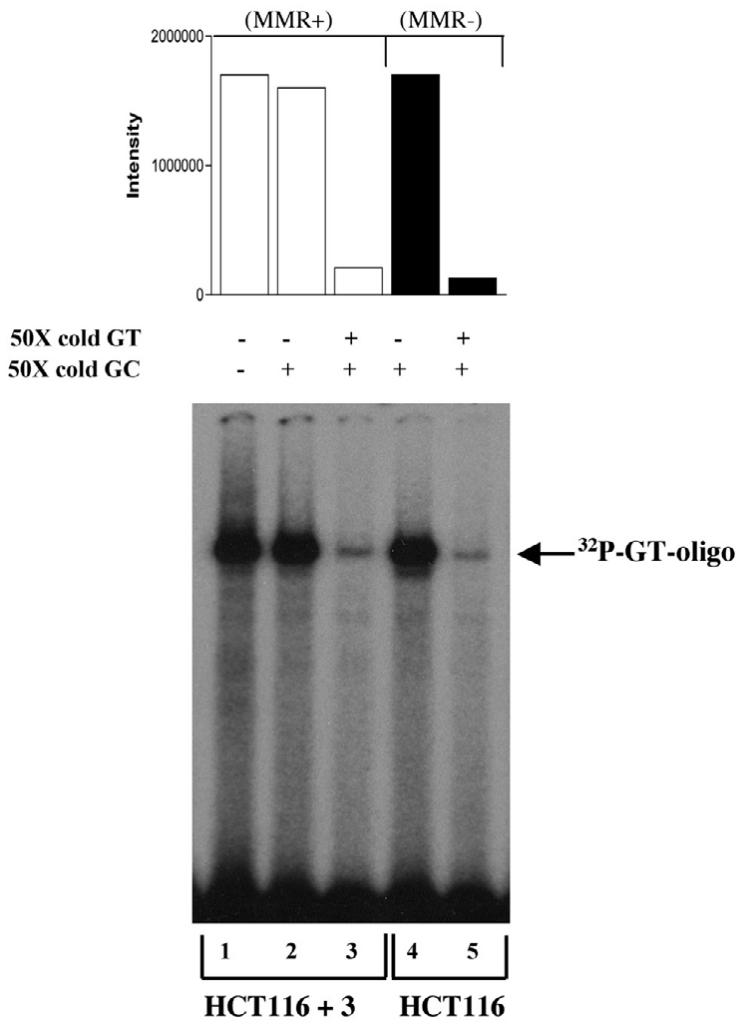
*Comparison of binding avidity of G:T-containing oligomer by MMR proficient (HCT 116 + Ch. 3) and MMR deficient (HCT 116; hMLH1^-/-^) nuclear extracts*. Equal aliquots of nuclear extracts from HCT 116 + Ch. 3 (lanes 1–3) or HCT 116 (lanes 4–5) were incubated with equal cpm of [^32^P]-G:T-oligo (69-mer) and 50X molar excess cold (unlabeled) oligo as indicated.

The HCT116 cell line completely lacks both 3' and 5' nick-directed MMR, putatively due to lack of hMLH1 expression, although these cells normally express hMSH2 and hMSH6. To determine if hMutSα recognition and binding of a mismatch at the H-*ras *codon 12 hot spot might undergo alteration within HCT116 cells, in conjunction with the lack of hMutLα, binding competition experiments were performed. Figure 2 is a comparison of the amount, or 'avidity', of hMutSα binding affinity within MMR proficient (HCT116 + Ch. 3) and within MMR deficient (HCT116) nuclear extracts. These gel shifts demonstrate virtually identical mismatch specific binding avidity of hMutSα to [^32^P]-G:T-oligo. As well, 50X molar excess of unlabeled G:T-oligo, which does not completely inhibit hMutSα gel shift of the radioactively labeled mismatch, demonstrates a similar degree of competitive inhibition of mismatch specific binding in both MMR proficient and MMR deficient nuclear extracts (Fig. 2, lanes 3 and 5). These results provide strong evidence that there is no discernable difference in hMutSα binding avidity to a G:T mismatch at H-*ras *codon 12, despite that HCT116 + Ch. 3 is MMR proficient and HCT116 lacks expression of hMutLα and is MMR deficient.

### Comparison of competitive inhibition of hMutSα binding avidity to G:T versus G:A mispairs at H-ras codon 12

**Figure 3:**
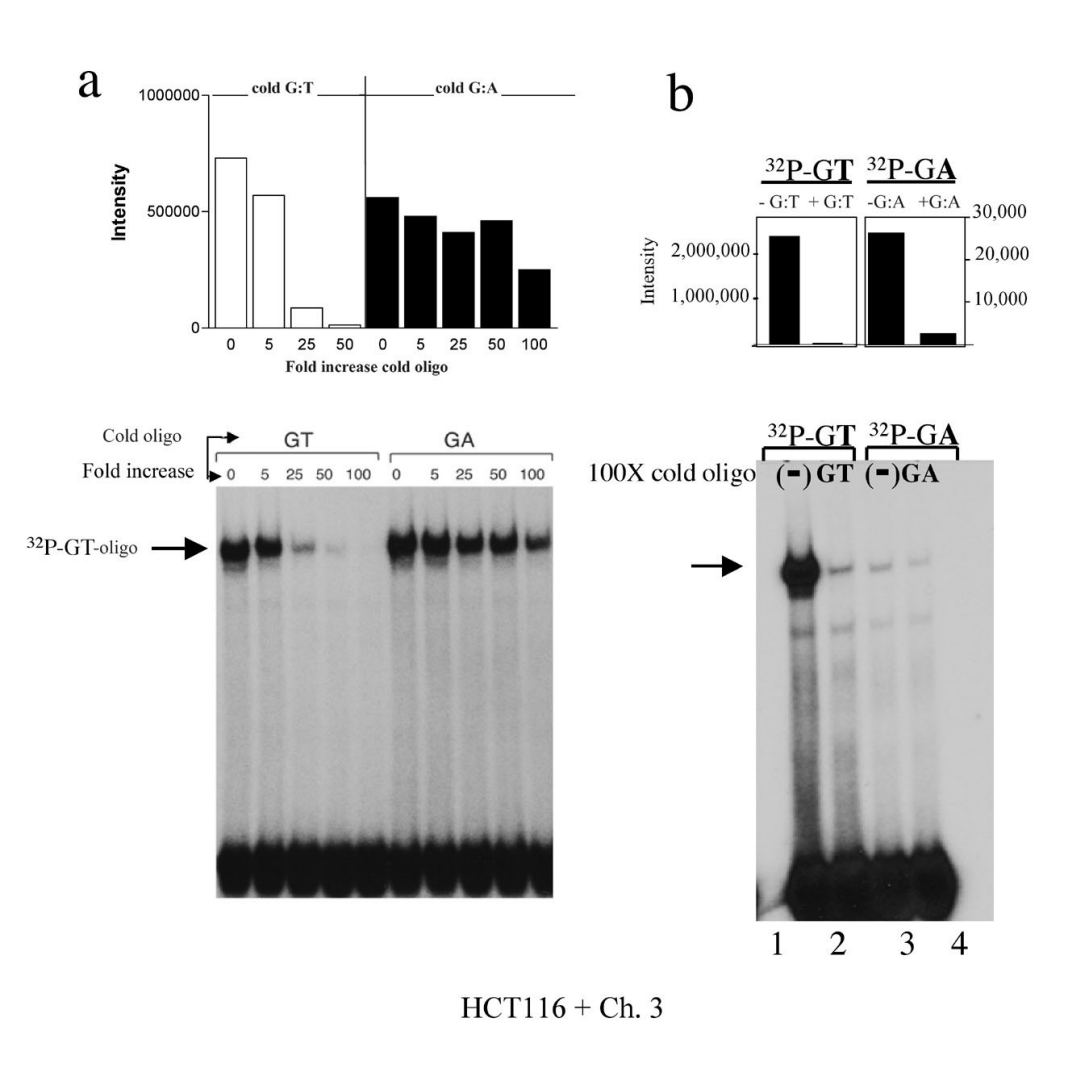
*Binding avidity of hMutSα to G:T versus G:A at H-ras codon 12 *(a) Equal cpm of [^32^P]-G:T-oligo (69 mer) were incubated with equal concentrations of nuclear extract (HCT116 + Ch. 3) in the presence of increasing concentrations of unlabeled (cold) G:T- or G:A-containing oligo, and 100X cold G:C oligo. (b) Equal aliquots of nuclear extract were incubated for 2.5 hours (versus 30 minutes) with equal cpm of each [^32^P]-G:T- or [^32^P]-G:A-oligo alone (lanes 1 and 3), or also with respective 100X molar excess cold G:T or G:A oligo (lanes 2 and 4). Bar graphs are densitometry results of corresponding radioactive band intensities.

We have previously determined that the G:A mismatch at H-*ras *codon 12 is accurately repaired back to G:C much less frequently than G:T [37]. In addition, we have previously demonstrated that recognition and binding of this poorly repaired mismatch is also very weak within HeLa nuclear extracts [23]. We therefore asked if competitive inhibition by several different concentrations of these mismatches would reveal more specific mechanisms of these intriguing differences at this same location. Firstly, using increasing concentrations of unlabeled G:T oligo as a competitor for [^32^P]-G:T-oligo, we observe the expected highly competitive inhibition of the radioactive G:T mismatch gel shift band in the presence of as little as 25X cold G:T-oligo (Figure 3a; compare cold G:T fold increase lanes "0" up through "100"). In comparison, the radioactive G:T-oligo gel shift band is much less competitively inhibited by increasing concentrations of cold G:A oligo (Figure 3a; compare cold G:A fold increase lanes "0" up through "100"), further confirming the observed preference of hMutSα for a G:T mismatch as compared to a G:A mismatch at the H-*ras *codon 12 hot spot of mutation. Comparison of the densitometric band intensities reveal that even 25X cold G:T oligo can successfully decrease hMutSα binding to radioactive G:T by 80%, but that 25X cold G:A decreases hMutSα binding to radioactive G:T by only 10%.

An alternate approach to more precisely define the extent of this phenomenon was to measure competitive inhibition of radioactively labeled G:A-oligo (rather than of the avidly bound [^32^P]-G:T-oligo) by incubation with unlabeled G:A-oligo, to determine if equal *ratios *of competitive binding might occur for G:T and G:A, despite the very different avidities of hMutSα for each mismatch. This would provide an indication of equal recognition of each type of mismatch by hMutSα, despite unequal binding avidity for these two mismatches. For these experiments, it was necessary to extend incubation periods from 30 min. to 2.5 hr, as sufficient concentrations of gel shifted bands can be detected with the G:A oligo only after extended incubation (unpublished observations). As expected, binding of hMutSα to [^32^P]-G:A-oligo is approximately 100 fold weaker in intensity than for [^32^P]-G:T-oligo after the extended incubation (Figure 3b; compare "Intensity" of lanes 1 and 3 on densitometric graph). However, 100X molar excess of cold G:A-oligo competitively inhibits MMR protein binding to [^32^P]-G:A-oligo by 89%, which is similar to the same concentration of cold G:T-oligo competitive inhibition of MMR protein binding to [^32^P]-G:T-oligo (97%), as determined by comparison of the densitometric intensity of each band. All of the above experiments have been repeated with similar results.

### MMR at H-ras codon 12 hot spot of mutation

Table 1 contains the results of a MMR assay designed to score nick-directed MMR as correct, incorrect or unrepaired [39]. Positive control experiments demonstrate that MMR at the H-*ras *codon 12 sequence within the pUC19 vector is nick-directed, yielding a high degree of correct repair by MMR proficient *E. coli *(DH5α); G:T → G:C = 95%, G:A → G:C = 78%, in agreement with our previously published results using a different plasmid vector [23]. Additionally, inserting the same H-*ras *69 mer into a pUC18 vector to determine effects of reverse orientation of the oligo and 'nicked' strand resulted in an almost identical frequency of correct repair at codon 12; G:T → G:C = 100%, G:A → G:C = 78%. These results confirm that the mismatch is recognized and repaired by nick-directed bacterial MMR and that there is no strand bias for MMR during high copy number bacterial replication of this plasmid. Background repair after direct transformation into NR9161 E. coli (MMR deficient) was consistently between 41–44% for both mismatches (data not shown), and is favorably comparable to an average of 50 – 60% background repair efficiency by this and other strains of MMR deficient E. *coli *(personal communication with Roel Schaaper).

Results within Table 1 were determined by incubating plasmids containing a G:T or G:A mismatch at the H-*ras *codon 12 middle base pair location containing a 5' nick on the "T" or "A" strand (as described in methods) with MMR proficient (HCT116 + Ch. 3) or MMR deficient (HCT116) nuclear extracts. Efficiency of correct, incorrect, and total (correct + incorrect) MMR for each mismatch was subsequently calculated (as described in methods) [39]. Surprisingly, *total repair *of the G:A mismatch (30%; G:A → G:C + T:A) was almost twice as high as for G:T (18.2%; G:T → G:C + A:T). This phenomenon was consistently observed within the several different experiments required to obtain the total results depicted in Table 1, and was statistically significant (p < 0.05). In direct contrast, *correct *(nick-directed) repair of G:A → G:C (60%) was significantly low when compared to *correct *(nick-directed) repair of G:T → G:C (96%) (p < 0.005). Inadvertent nicks in the "G" (correct) strand of the H-*ras *69 mer or pUC plasmid during preparation could not have contributed to the increased total, or increased incorrect repair (G:A → T:A) results for the G:A mismatch because the efficiency of incorrect repair would then be similar for both the G:T and the G:A mismatch. Instead, MMR results within Table 1 were found to be consistently different for the two mismatches, and are the compiled results of several different mismatch-containing plasmid preparations and subsequent MMR assays. In addition, MMR proficient *E*. *coli *nick-directed repair results are consistently high, for both mismatch-containing plasmid preparation subsequently used for each nuclear extract MMR assay (data not shown). As well, MMR deficient E. *coli *repair ratios (correct to incorrect) are 1:1 after transformation of unmethylated plasmids (prepared by replication in GM2929; *E. coli dam*^-^*dcm*^-^). In combination, all of the above control experiments demonstrate a lack of any significant unintentional misrepair events on either strand of DNA, for either site-specific mismatch plasmid preparation. Therefore, the results within Table 1 suggest that increased total repair (correct + incorrect) combined with increased incorrect repair of G:A, as compared to the decreased total repair and increased correct MMR repair of G:T, may result in increased mutational events when a G:A mismatch occurs at this oncogenic site. Somewhat surprisingly, MMR deficient nuclear extracts did not repair either mismatch at codon 12 above background, indicating a complete lack of alternate DNA repair activity that can correct either mismatch at this oncogenic site. Therefore these results indicate that the observed repair efficiencies in MMR proficient nuclear extracts are due solely to MMR activity, rather than to a combination of MMR and other DNA repair pathways. Alternatively, it is possible that the methods used for nuclear extract preparation or the *in vitro *MMR assay might cause an artifactual decrease in the activity of other DNA repair pathways.

## Discussion and conclusions

Previous investigations of ours have revealed significantly decreased nick-directed G:A → G:C repair at codon 12 of H-*ras*, as compared to G:T → G:C repair at this location [23,37]. Decreased MMR of G:A has also been observed within different sequences by other investigators, although molecular mechanisms for these differences remain obscure [12]. The current investigation was undertaken to determine key steps differentiating G:T and G:A repair pathways at the H-*ras *oncogenic hot spot within the nuclear environment. Our results suggest that, firstly, the MMR pathway is the primary, if not the only, DNA repair pathway that can recognize and is subsequently responsible for the repair of both mismatches at this oncogenic location within the cell. Secondly, although 'recognition' of either G:T or G:A by hMutSα is likely equal, and is essential for initiation of MMR, the avidity – or strength – of hMutSα binding to either mismatch is not equal, and may play a significant role in the decision of whether to activate nick-directed *correct *MMR, or the activation of an alternate response pathway. In support of this concept, and in correlation with weak hMutSα binding to G:A, we have observed significantly decreased nick-directed (correct) MMR of G:A → G:C (60%), as compared to nick-directed MMR of G:T → G:C (96%). We have however, consistently measured almost twice the efficiency of *total *repair of G:A (to either G:C or T:A) as compared to total G:T repair (to G:C or A:T) at this oncogenic site. This phenomenon appears to be due to increased non nick-directed *incorrect *repair of G:A → T:A (rather than to replication without repair, which results in a mixture of G:C and T:A).

This raises the possibility that (a) initial recognition of a mismatch is essential but separable from (b) differential binding of MMR proteins to specific mismatches, which in turn is directly correlated either with nick-directed *correct *MMR, or with alternate events other than nick-directed MMR. In support of this concept, Wang, et. al. has recently demonstrated by atomic force microscopy that *E. coli *MutS-DNA complexes exist in two conformations [40]. The initial recognition of a mismatch by MutS results in a localized kink in the DNA conformation and is termed the initial recognition complex (IRC). The second step is required for MMR and is a further conformational change in which the localized kink in the DNA becomes unbent. This is called the ultimate recognition complex (URC) and may be the conformation required for ATP activation and subsequent MMR activity. It is also possible that either differential binding kinetics (not measurable by gel shift), or very low avidity of binding or a different molecular binding mechanism (undetectable by gel shift) may contribute to subsequent cellular events specific to the G:A mismatch. These possibilities are currently being investigated. An additional hypothesis has been proposed by Junop, et. al., placing *E. coli *MutS in the role of 'authorizing' different repair events [41]. Also, it is now well documented that while hMutSα recognizes DNA damage other than mismatches, the MMR pathway does not appear to play a direct role in the repair of these damaged bases, but rather is associated with initiation of cell cycle and/or apoptotic events and therefore is described as a "sensor of genetic damage" within this context. The molecular mechanisms contributing to the various cellular activities associated with the well documented hMutSα recognition and differential binding to different DNA structures is not yet clear. Although there does not appear to be a consistent physical size or shape of hMutSα recognizable structures that trigger different pathways of cellular activity, DNA sequence context does appear to play a role [9,12].

**Figure 4:**
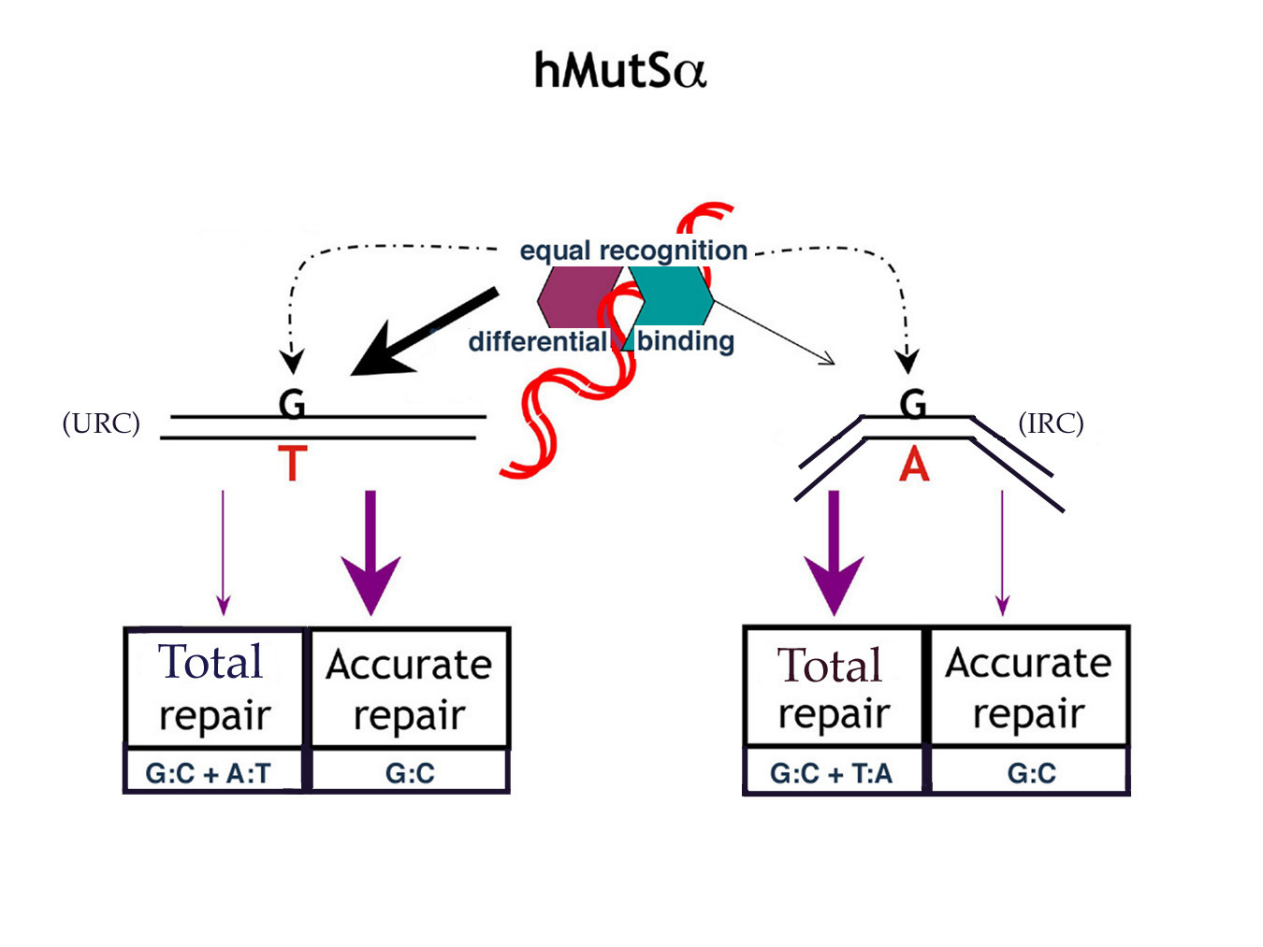
*Repair model of hMutSα MMR*. Model of hMutSα initial recognition (equal) of G:T and G:A, followed by repair (differential) of DNA containing a G:T or a G:A mismatch at the H-*ras *codon 12 location. hMutSα recognizes and forms an initial recognition complex (IRC) with both mismatches equally, and then binds more strongly to G:T, perhaps by undergoing an additional conformational step to the ultimate recognition complex (URC), which does not occur with G:A [40]. This results in more accurate repair of G:T, but more frequent total repair of G:A. See text for further discussion.

Figure 4 is the model summarizing our hypothesis. This concept is compatible with our current set of experimental data, and with the recently described two-step conformational alteration of the *E. coli *MutS-DNA recognition complex [40]. Briefly, hMutSα appears to equally recognize both G:T and G:A mismatches at the codon 12 hot spot of mutation, but binds more avidly to G:T, or the "URC" conformation. The stronger, or alternate conformational, binding of hMutSα to G:T results in increased accuracy of MMR, but may decrease overall repair efficiency. The less avidly bound G:A mismatch, or the "IRC" conformation, is repaired with increased total efficiency as compared to G:T, but accuracy is sacrificed.

These results agree with, and build upon our previous experimental results, and also agree with the presence of specific mutations predominantly found in human tumors [23,24,37]. It has now been demonstrated by several different investigators that, although any base pair other than G:C at the H-*ras *codon 12 location is activating, the majority of human tumors containing mutations at this site are G:C → T:A transversions [42-44]. Our observed increased *overall *MMR of G:A, combined with a high ratio of *incorrect *MMR of G:A → T:A at this oncogenic location correlates well with the predominant G**G**C → G**T**C (Gly → Val) *ras-*activating mutation found in a high percentage of human tumors [42,44,45]. Thus the demonstration of a significant increase in the frequency of both total repair and incorrect repair of the G:A mismatch at the H-*ras *codon 12 oncogenic hot spot of mutation, combined with a complete lack of rescue by other DNA repair pathways, is biologically relevant.

## Methods

### Nuclear extracts, oligonucleotides, and site-specific mismatched plasmids

Human colorectal carcinoma cell lines HCT116 and HCT116 + Ch. 3 were cultured in Iscove's Modified Dulbecco's Medium supplemented with 10% fetal bovine serum (FBS) at 37°C, 5% CO_2_. HCT116 + Ch. 3 cell line also received 0.4 mg/ml Geneticin (G418). Both HCT cell lines were kind gifts of C. Richard Boland; UCSD. Nuclear extracts were prepared as described previously [46]. Synthetic oligonucleotides of 69 bases containing the coding strand sequence 5'-*AATTC*ACGGAATATAAGCTGGTGGTGGTGGGCGCCG**G**CG GTTGGGCAAGAGTGCGCTGACCATCCAG*G*-3', as well as complementary noncoding oligomers, were obtained from Operon (Alameda, CA). The above 69 mer oligo is a portion of the coding sequence of human H-*ras *DNA. The bolded underlined **G**represents H-*ras *codon 12 middle G position, plus an additional 30 bases of H-*ras *sequence both 5' and 3' of codon 12, with an *Eco R1 *site 5' and a *Bam H1 *site 3' (restriction enzyme recognition nucleotides in italics). The wild type sequence (coding strand containing codon 12 middle G) was 5'-phosphorylated. Mismatch containing noncoding strand sequences (either T or A opposite codon 12 middle base G) were not phosphorylated, thus providing a 5' nick in the strand containing the incorrect base after ligation into the pUC19 plasmid. All other reagents were purchased from Sigma unless otherwise noted.

### Preparation of site-specific mismatched oligonucleotides and plasmids

Complementary 69 mer oligos containing the wild type sequence at codon 12; middle base (coding strand G) and one mismatched base (noncoding strand T or A), as described above, were annealed in an equimolar ratio at a final concentration of 0.2 μg DNA per μl of annealing buffer (1 mM Tris-HCL, 1 mM MgCl_2_, pH 7.5). Ligation of annealed mismatched oligos to *Eco R1/Bam H1 *digested pUC19 DNA was accomplished at a 20:1 molar ratio of 69 mer to pUC19 DNA (~3–6 ng DNA per μl of ligation solution), using T4 ligase, per manufacturer's recommendations (Invitrogen Corp.; Carlsbad, CA). The pUC19 vector used for ligation of mismatch-containing H-*ras *oligo for subsequent measurement of MMR within nuclear extracts was grown in GM2929 (*E. coli dam*^-^*dcm*^-^). The pUC19 and pUC18 plasmids used as positive controls for correct bacterial mismatch repair were grown in DH5α (*E. coli dam*^+^).

### Electrophoretic gel mobility shift assays

Gel shift assays were performed using nuclear extracts from either HCT116 or HCT116 + Ch. 3 cells and [^32^P]-dATP-labeled-69 mer duplexes by a fill-in reaction, using [α-^32^P]-dATP and Klenow polymerase, per manufacturer's protocol (Invitrogen). Each nuclear protein-DNA binding assay was performed using 0.8 – 1.0 × 10^5 ^cpm homoduplex or heteroduplex 69 mer and 5–10 μg protein from nuclear extract solution in an equal volume of 2X gel shift reaction buffer, resulting in a final concentration of 1 mM MgCl_2_, 0.5 mM EDTA, 0.5 mM DTT, 50 mM NaCl, 10 mM Tris-HCL, pH 7.5, 0.1 mg/ml poly [dI:dC], 5 mg/ml BSA, 6% glycerol, in a final volume of 15 – 20 μl. In addition, each reaction contained 100-fold molar excess (100X) unlabeled homoduplex 69 mer, unless otherwise noted. Incubations were for 30 min. at 37°C, unless otherwise noted. Gel shifts of each reaction were electrophoresed at 20 mA within a 4.8% nondenaturing acrylamide gel in 0.5 × TBE buffer, at room temperature. Radioactively labeled oligomers were visualized by autoradiography of the dried gel.

### Site-specific mismatch repair assay within human nuclear extracts

An *in vitro *mismatch repair assay was performed essentially as described by Thomas et. al. [39], except for modifications as described below. For each reaction, 14 fmols of pUC19 plasmid containing site-specific mismatched H-*ras *69 mer, with a nick on the same strand and 36 bases 5' of the incorrect base, was incubated with 50 μg of HCT116 + Ch. 3 or HCT116 nuclear extract in a final volume of 25 μl containing 30 mM HEPES buffer, pH 7.9, 100 μM each of dATP, dCTP, dGTP and dTTP, 200 μM each of CTP, GTP and UTP, 4 mM ATP, 40 mM creatine phosphate, 100 ng/μl creatine phosphokinase, 0.5 mM DTT, and 7 mM MgCl_2_. Negative control experiments were performed in the above solution without nuclear extract. Each reaction was incubated at 37°C for one hour, as described [39]. Plasmid DNA containing the H-*ras *insert was recovered using Wizard SV Miniprep System per manufacturer's directions (Promega; Madison, WI), and subsequently used to transform E.*coli *strain NR9161 (*MutL*^-^). In addition, DH5α (MMR proficient E. *coli*) were transformed directly with the ligated and nicked plasmid as a positive control for correct mismatch repair [37]. Subsequently, plasmid DNA was purified from ampicillin-resistant bacterial colonies and digested with *Nae I*, which recognizes a single restriction digestion site unique to the wild type H-*ras *sequence at codon 12 middle base pair (G:C) [23]. After electrophoresis in 1% agarose, banding patterns of resulting DNA fragments were analyzed to score plasmids as having correct, incorrect, or no repair [23,37,38].

MMR efficiency of each human nuclear extract assay, above MMR deficient bacterial background repair, was determined by the following equations [39]:

Total repair efficiency = 100 × (1 - [fraction of unrepaired plasmids incubated with human nuclear extract results / fraction of unrepaired untreated plasmids from direct transformation of NR9161]).

Correct repair efficiency = 100 × {(fraction of correctly repaired incubated with human nuclear extract) - [(fraction of correct repair by NR9161 direct transformation) × (1 - fraction of total repair efficiency)]}.

Incorrect repair efficiency = Total repair efficiency - Correct repair efficiency.

Statistical comparisons of G:T and G:A results were conducted by a non-parametric equivalent to the Student's t-test for differences between proportions. Statistical significance was indicated for those comparisons with a P < 0.05 assuming a 2-tailed distribution [47].

## Authors' contributions

ME designed and carried out the MMR assay studies and helped draft the manuscript. HH designed and carried out all other experiments. AS performed all densitometry measurements and helped draft the manuscript. MF and SB helped carry out the MMR assays. KW conceived of the study, participated in its design and coordination and wrote the final manuscript.

## Figures and Tables

**Table 1:** Mismatch repair at an oncogenic site within MMR proficient and MMR deficient human nuclear extracts.

	**HCT 116 + Ch. 3; H-*ras *codon *12 *middle G:C**		**HCT 116; H-*ras *codon *12 *middle G:C**	
	
	**G:T**	**G:A**	**G:T**	**G:A**
*Total repair^a ^(correct & incorrect)	**18.2 %**	**30 %**	∅^b^	∅
				
**Correct repair^a ^(% of total)	(96%)	(60%)	∅	∅
**Incorrect repair^a ^(% of total)	(4%)	(40%)	∅	∅
				
**Total # analyzed**	99	100	78	91

^a ^Repair efficiency above background for each mismatch at H-*ras *codon 12 by each human nuclear extract was determined by the following equations [39].

***Total repair efficiency***= 100 × (1 - [fraction of nonrepaired mixture incubated with human nuclear extract results / fraction of nonrepaired results of direct transformation of NR9161]).

***Correct repair efficiency***= 100 × {(fraction of correctly repaired incubated with human nuclear extract) - [(fraction of correct repair by NR9161 direct transformation) × (1 - fraction of total repair efficiency)]}.

***Incorrect repair efficiency***= Total repair efficiency - Correct repair efficiency.

^b ^No repair detected above background.

* Designates statistical difference between HCT116 + Ch.3 G:T and G:A repair at P < 0.05.

**Designates statistical difference between HCT116 + Ch.3 G:T and G:A repair at P < 0.005.
